# Comparison of Functional Proteomic Analyses of Human Breast Cancer Cell Lines T47D and MCF7

**DOI:** 10.1371/journal.pone.0031532

**Published:** 2012-02-24

**Authors:** Juliette Adjo Aka, Sheng-Xiang Lin

**Affiliations:** 1 Laboratory of Endocrinology and Genomics, CHUL Research Center (CHUQ), Laval University, Québec City, Québec, Canada; 2 Department of Molecular Medicine, Faculty of Medicine, Laval University, Québec City, Québec, Canada; University of Medicine and Dentistry of New Jersey, United States of America

## Abstract

T47D and MCF7 are two human hormone-dependent breast cancer cell lines which are widely used as experimental models for *in vitro* and *in vivo* (tumor xenografts) breast cancer studies. Several proteins involved in cancer development were identified in these cell lines by proteomic analyses. Although these studies reported the proteomic profiles of each cell line, until now, their differential protein expression profiles have not been established. Here, we used two-dimensional gel and mass spectrometry analyses to compare the proteomic profiles of the two cell lines, T47D and MCF7. Our data revealed that more than 164 proteins are differentially expressed between them. According to their biological functions, the results showed that proteins involved in cell growth stimulation, anti-apoptosis mechanisms and cancerogenesis are more strongly expressed in T47D than in MCF7. These proteins include G1/S-specific cyclin-D3 and prohibitin. Proteins implicated in transcription repression and apoptosis regulation, including transcriptional repressor NF-X1, nitrilase homolog 2 and interleukin-10, are, on the contrary, more strongly expressed in MCF7 as compared to T47D. Five proteins that were previously described as breast cancer biomarkers, namely cathepsin D, cathepsin B, protein S100-A14, heat shock protein beta-1 (HSP27) and proliferating cell nuclear antigen (PCNA), are found to be differentially expressed in the two cell lines. A list of differentially expressed proteins between T47D and MCF7 was generated, providing useful information for further studies of breast cancer mechanisms with these cell lines as models.

## Introduction

Breast cancer is the most frequent cancer affecting women. The malignancy accounts for about 1 in 10 cancers in the world and is diagnosed in one million women each year [1], [2]. In North America (United States and Canada), breast cancer is the second most frequent cause of cancer death in women, after lung cancer, and the leading cause of cancer death among those aged 20–59 years old [3], [4]. After increasing through the 80 s and 90 s, breast cancer incidence rates fortunately decreased by 3.5% per year from 2001 to 2004 and the mortality rate decreased by 1.9% per year in the United States between 1998 and 2006 [3], [4]. This reflects an improvement in the diagnosis and treatment of the disease, but this cancer nonetheless remains of prime importance.

Human breast cancer cell lines provide an excellent platform for breast cancer research in tumor progression and treatment. T47D and MCF7 are two human hormone-dependent breast cancer cell lines which are widely used as experimental models for breast cancer studies. The two cell lines were generally used for both the *in vitro* (in cell culture) and *in vivo* (tumor xenograft in nude mice) analyses of gene and protein function and inhibitor efficacy assessment [5]–[7]. They were both originally derived from a metastatic site of pleural effusion (ATCC, www.atcc.org) and express estrogen receptors. Several proteins and enzymes that are involved in cell proliferation and in cancer development were identified in these cell lines by proteomic studies [8]–[10]. Although these studies reported the proteomic profiles of each of these cell lines, until now, no study had established their differential protein expression profile. Using a proteomic approach including two-dimensional (2-D) gel electrophoresis and mass spectrometry (MS) analyses, we establish here the proteomic differences between the T47D and MCF7 cell lines.

## Methods

### Cell culture

T47D and MCF7 cells were obtained from the American Type Culture Collection (ATCC, Manassas, VA). MCF7 cells were maintained in DME low glucose medium supplemented with 1 nM β-estradiol (β-E2). T47D cells were propagated in DME high glucose medium containing 7.5 mg/L bovine insulin (Sigma, Oakville, Ontario, Canada). Both cell types were cultured in phenol red-free media containing 10% fetal bovine serum (FBS) and incubated at 37°C in a humidified atmosphere of 95% air and 5% CO_2_.

### Generation of protein extracts for proteomics analysis

MCF7 and T47D cells were cultured in T75 flasks in complete growth medium. After three passages, cells were plated in 100×2 cm2 dishes and cultured until reaching 80–90% confluence. Cells were washed two times with cold PBS 1×, scraped with a policeman in 1.2 mL PBS, collected in an eppendorf and centrifuged at 3000 rpm for 5 min. The cell pellets were resuspended in 500 µl lysis buffer T8 (7 M urea, 2 M thiourea, 3% CHAPS, 20 mM DTT, 5 mM TCEP, 0.5% IPG buffer pH 4–7, 0.25% IPG buffer pH 3–10) containing 50 mM tris-HCl pH 8.8, 1 mM PMSF and 1% protease inhibitors cocktail (EMD Chemicals, Gibbs-town, NJ). Protein samples were precipitated using 2-D Clean-Up Kit (GE Healthcare, Piscataway, NJ) and resolubilized in T8 buffer. Protein samples included three independent biological replicates (coming from three independent cell culture experiments), representing total proteins from each cell line (MCF7 and T47D) for a total of six samples. The protein concentrations were determined using the 2-D Quant Kit (GE Healthcare).

### Two-dimensional gel electrophoresis

For the first dimension, 200 µg total protein samples from MCF7 and T47D cell lines were loaded onto 24-cm pH 4–7 Immobilized pH gradient (IPG) strips (Immobiline DryStrips; GE Healthcare). Strips were rehydrated for 10 hours at 30 volts and isoelectric focusing was performed on an IPGphorII IEF system (GE Healthcare). For the second-dimension SDS-PAGE, focused Immobiline DryStrips were equilibrated twice for 15 min in an equilibration buffer (50 mM tris-HCl pH 8.8, 6 M urea, 30% glycerol, 2% SDS, trace of bromophenol blue) containing 10 mg/mL DTT for the first equilibration and 25 mg/mL iodoacetamide for the second one. Immobiline DryStrips were then transferred onto the surface of a 12% acrylamide gel and sealed using 0.5% agarose. Gels were run in Ettan DALT*twelse* system (GE Healthcare) in a standard tris-glycine SDS-PAGE buffer at 40 mA/gel and 15°C until the tracking dye reached the end of the gel. Three independent protein samples coming from three independent cell culture experiments were run for each cell line. Gels were fixed overnight in 40% methanol, 7% acetic acid, stained with Sypro Ruby (Invitrogen, Burlington, Ontario, Canada) and scanned with the ProXpress CCD scanner (PerkinElmer, Waltham, MA). The 2-D gel electrophoresis was performed at the Proteomic platform of the Infectious Disease Research Center (Quebec, Canada).

### Two-dimensional gel image analysis

Protein spot detection, spot matching and semiquantitative statistical analysis were performed using the Progenesis software version PG240 (Nonlinear Dynamics, Durham, NC). For each cell line, three different gel images were analysed and a corresponding synthetic image reference was obtained. After computer matching, detected spots and spot matches were manually edited for more accuracy. A spot had to be present in at least two of the three replicate gels to be considered in the analysis. The detection of protein spots differentially expressed was performed using the *t*-test and INCA volume and proteins that were differentially expressed 2-fold or higher were considered significant. 40 protein spots that were differentially expressed were selected and were excised from Sypro Ruby-stained 2-D gels using a ProXcision robot (PerkinElmer) and sent for MS analysis.

### Mass spectrometry and protein identification

MS experiments were performed by the Proteomics platform of the Eastern Quebec Genomics Center (Quebec, Canada). Protein spots were washed with water and tryptic digestion was performed on a MassPrep liquid handling robot (Waters, Milford, MA) according to the manufacturer's specifications and to the protocol of Shevchenko et al [11] with the modifications suggested by Havlis et al [12]. Peptide samples (an aliquot of the digested proteins) were separated by online reversed-phase (RP) nanoscale capillary liquid chromatography (nanoLC) and analyzed by electrospray mass spectrometry (ES MS/MS). The experiments were performed with a Thermo Surveyor MS pump connected to a LTQ linear ion trap mass spectrometer (ThermoFisher, San Jose, CA) equipped with a nanoelectrospray ion source (ThermoFisher). Peptide separation took place on a PicoFrit column BioBasic C18, 10 cm×0.075 mm internal diameter (New Objective, Woburn, MA) with a linear gradient from 2–50% solvent B (acetonitrile, 0.1% formic acid) in 30 minutes, at 200 nL/min (obtained by flow-splitting). Mass spectra were acquired using a data dependent acquisition mode using Xcalibur software version 2.0. Each full scan mass spectrum (400 to 2000 m/z) was followed by collision-induced dissociation of the seven most intense ions. The dynamic exclusion (30 sec exclusion duration) function was enabled, and the relative collisional fragmentation energy was set to 35%.

All MS/MS samples were analyzed using Mascot algorithm (Matrix Science, London, UK; version Mascot) and the Uniref100_14_0_Homo_sapiens_9606 database (version with 89892 entries). Mascot was searched with a fragment ion mass tolerance of 0.50 Da and a parent ion tolerance of 2.0 Da. Iodoacetamide derivative of cysteine was specified as a fixed modification and oxidation of methionine was specified as a variable modification. Two missed cleavages were allowed. Scaffold (version Scaffold_2_01_02, Proteome Software Inc., Portland, OR) was used to validate MS/MS based peptide and protein identifications. The protein identification cut off was set at a confidence level of 95% (MASCOT score >33) with at least two peptides matching to a protein. Proteins that contained similar peptides and could not be differentiated based on MS/MS analysis alone were grouped to satisfy the principles of parsimony.

### Functional analysis of the identified proteins

From each spot, only proteins identified with a probably higher than 95% and with at least two matched unique peptides were considered in the analysis, except for the proteins keratins which were not considered for the analysis. The experimental molecular weight and isoelectric point of each identified protein were determined based on the location of the original spot on the 2-D gel using the Progenesis software. The Uniprot data base (www.uniprot.org) was used to search the function/biological process and the subcellular location of each identified protein. Search in the literature (Pubmed) was used when necessary to complete the information about the function and subcellular location.

### Quantitative real-time RT-PCR

Total RNAs were isolated from T47D and MCF7 cells using Trizol Reagent (Invitrogen) in 6-well plates and treated with DNase 1. RNA samples for Q-RT-PCR analyses comprised two biological repetitions for each cell line. The measures of mRNA levels of genes were carried out as previously described [7], [13] with *Atp5o*, *Hprt1* and *G6PD* genes used as internal controls. The procedures were performed at the Q_RTPCR Platform service at CHUQ-CHUL Research Center (Quebec, Canada). The mRNA levels were expressed as thousand of mRNA copies/µg total RNA and SDs were <10% of duplicates.

## Results

### The proteome comparison of T47D and MCF7 cells

**Figure 1:**
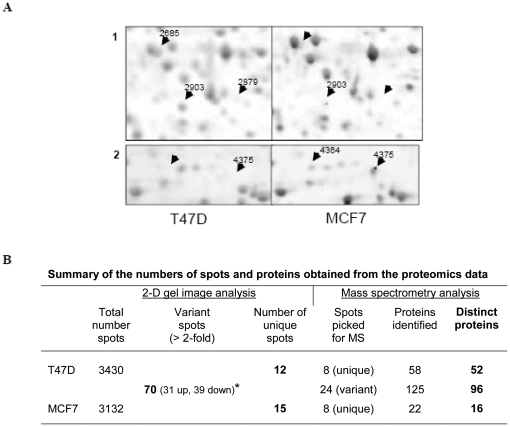
Proteomic analysis of T47D and MCF7 cells using 2-D gels and mass spectrometry. (A) Representative 2-D gel images for T47D and MCF7 cells showing some differentially expressed spots. The 2-D gels were scanned and the differentially expressed (2-fold or higher, *p*<0.05) proteins were detected using Progenesis software. Arrows indicate some identified protein spots picked for MS analysis. The numbers refer to the spot number listed in Table 1 and 2. Spot numbers 2685, 2879 and 4364 were unique in either cell line whereas spot numbers 2903 and 4375 were up-regulated in one cell line compared to the other cell line. 1 and 2 represent two different regions of the entire 2-D gel images. (B) Summary of the numbers of spots and proteins obtained from the 2-D gel and mass spectrometry analyses of T47D and MCF7. * Up-regulated (up) and down-regulated (down) proteins in T47D cell line as compared to MCF7 cell line.

To compare the proteomes of T47D and MCF7 cells, we performed 2-D gel analysis using total protein lysates of the two cell lines. The analysis was carried out on six 2-D electrophoresis gels made from three independent protein samples of each cell line. The Progenesis Discovery software package was used to carry out statistical comparative analyses of the proteomic profiles of T47D and MCF7 cells. The two cell lines displayed similar spot patterns, which allowed a good spot alignment (Figure 1A). T47D protein samples exhibited 298 supplementary spots compared to MCF7 (Figure 1B), suggesting that the former cells express a higher number of proteins than the latter. The proteomic analyses using the Progenesis software and a *t*-test (with a *p*-value<0.05) identified 97 significant differential spots as follows: 70 spots exhibited a variation≥2-fold, including 31 spots up-regulated and 39 spots down-regulated in T47D, whereas 12 and 15 spots were found unique to T47D and MCF7, respectively (Figure 1B). Some differentially expressed spots are shown in more detail in Figure 1A.

For the next step, MS identification, protein spots were selected among those uniquely and strongly (more than 3-fold difference) overexpressed, but also among those weakly (2 to 3-fold difference) up-regulated and well defined in each cell. The selection of weakly up-regulated spots was relevant to detect small proteomic differences between cells. A total of 40 spots were excised from Sypro Ruby-stained 2-D gels and were subjected to trypsin digestion. The resulting peptide fragments were analyzed by MS. Proteins with known UniProt accession numbers were identified in all the 40 spots. The numbers of proteins revealed by MS analysis are listed in Figure 1B. A total of 205 proteins were identified from these 40 spots, with a number of them found in multiple spots. For example, nitrilase homolog 2 was found in only one spot, the spot number 4364, while cathepsin D was found in several spots including spot numbers 2685 (Figure 1A). Consequently, distinct proteins amount to 164 with 52 and 16 proteins from spots unique to T47D and MCF7, respectively, and 96 proteins from differential spots. These results revealed that T47D and MCF7 cells present some significant differences in regard to their proteomes.

### Functional and subcellular protein categorizations

**Figure 2:**
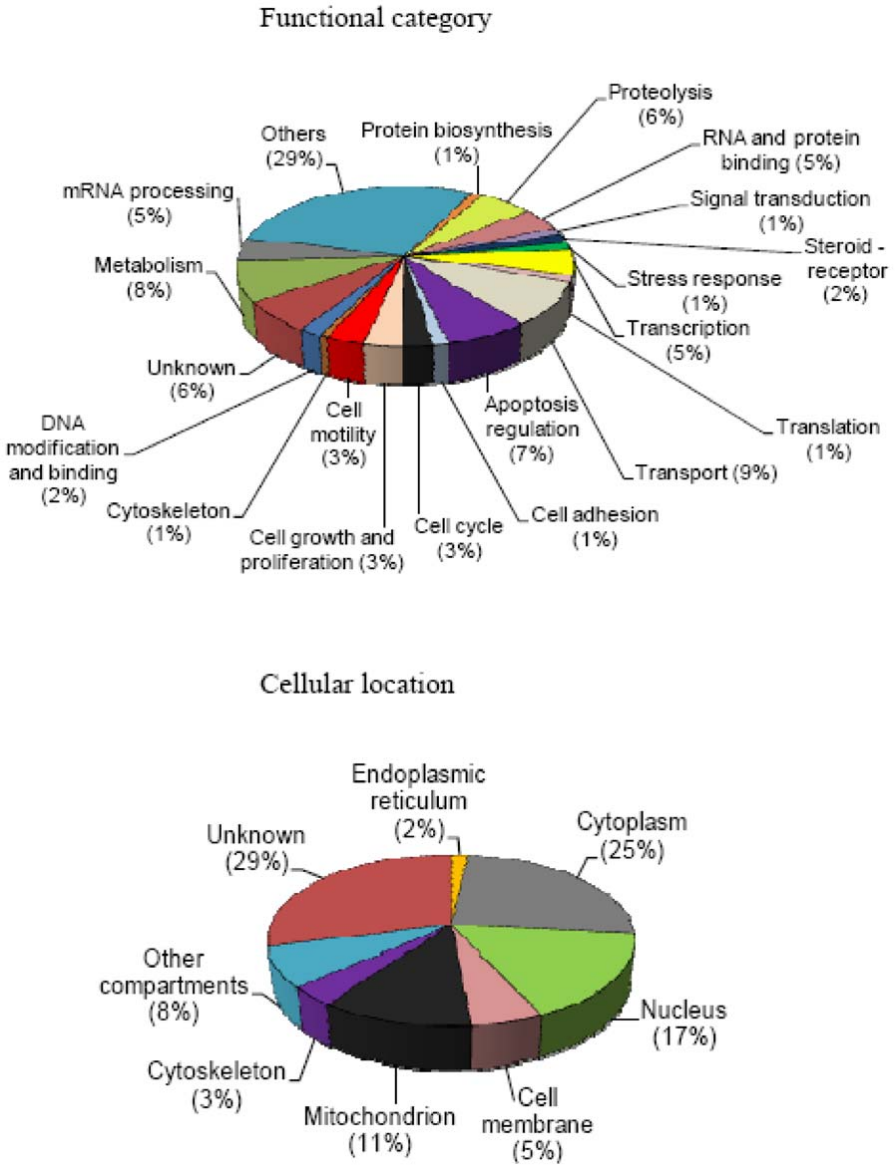
Functions and cellular locations of the differentially expressed proteins in T47D and MCF7 cells identified by the proteomics approach. The Uniprot database was used to generate the cellular location and the molecular function and/or biological process of each of the 164 non-redundant (distinct) proteins identified by mass spectrometry analysis as differentially regulated in T47D as compared to MCF7 cells.

Using the UniProt database at www.uniprot.org, we determined the functions and/or biological processes of each identified protein (Table 1, 2 and S1). Table 1 and Table S1 list the proteins found in spots up-regulated or unique to T47D as compared to the MCF7 cell line while Table 2 lists the proteins found in spots down-regulated in T47D or unique to MCF7. The spot from which each protein was identified, the spot fold-increase or fold-decrease in one cell line *versus* the other cell line, the protein name, the molecular weight, the isoelectric point, the number of unique peptides allowing the identification of each protein in the MS analysis, and the UniProt accession number of the protein, were mentioned. The information about the molecular function and/or biological process and subcellular location was found for most proteins. The repartition of each function and subcellular location are illustrated in Figure 2. From the 164 proteins identified by MS, 14 were principally implicated in transport, 13 in metabolism, 11 in apoptosis, 9 in proteolysis, 8 in transcription, 7 in mRNA processing and 7 in RNA and protein binding. Differentially expressed proteins are mainly located in cytoplasm and nucleus.

**Table 1:** Mass spectrometry identification of proteins in spots up-regulated in or unique to T47D as compared to MCF7 cell line.

Spot	FC	Description	UniProt number	MW exp/pred (kDa)	pIexp	Pep	Function description and/orbiological process
2810	3.0	Phosphoserine phosphatase	P78330	26/25	5.4	15	Serine biosynthesis
		Uncharacterized protein EIF4E (Fragment)	A8MX72	26/25	5.4	5	Protein biosynthesis
		cDNA FLJ76387…arginine/serine-rich 9…	A8K3M9	26/26	5.4	5	Nucleic acid binding
		Transmembrane emp24 domain-containing prot. 7	Q9Y3B3	26/25	5.4	5	Transport
		**Heat shock protein beta-1** ‡_(2873,2808,4439)_	P04792	26/23	5.4	3	Stress resistance, actin organization
		Peroxiredoxin-4	Q13162	26/31	5.4	2	Redox regulation of the cell
2490	6.1	P09493-3 Isoform 3 of P09493	P09493-3	32/33	4.6	38	Muscle contraction
		**Proliferating cell nuclear antigen** * ‡	P12004	32/29	4.6	16	DNA replication
2682	2.1	**Protein CDV3 homolog** *_(2658,2399)_	Q9UKY7	28/27	5.4	6	Cell proliferation
		N(G),N(G)-dimethylarginine dimethylaminohydrolase 2	O95865	28/30	5.4	5	Anti-apoptosis, arginine catabolism
		Prohibitin _(2674,4384)_	P35232	28/30	5.4	5	DNA synthesis, cell proliferation
2869	3.2	Glutathione S-transferase Mu 2	P28161	25/26	6.1	53	Metabolic process
		5′(3′)-deoxyribonucleotidase, cytosolic type	Q8TCD5	25/23	6.1	25	Nucleotide metabolism
		**17β-Hydroxysteroid dehydrogenase 10** *	Q5H927	25/26	6.1	6	Oxidoreductase - metabolism
		Protein-L-isoaspartate(D-aspartate) O-methyltransferase	P22061	25/25	6.1	4	Protein methylation, protein repair
2903	4.3	**Cathepsin B** * ‡	P07858	24/38	5.1	4	Tumor invasion and metastasis
		Heat shock protein 75 kDa, mitochondrial	Q12931	24/80	5.1	3	Chaperone
2399	3.0	Nicotinate-nucleotide pyrophosphorylase	Q15274	35/31	5.8	9	Pyridine nucleotide biosynthesis
		Malate dehydrogenase, cytoplasmic	P40925	35/36	5.8	4	Gluconeogenesis
		L-lactate dehydrogenase A chain	P00338	35/37	5.8	4	Glycolysis
		Farnesyltranstransferase	O95749	35/35	5.8	4	Isoprene biosynthesis
		Splicing factor, arginine/serine-rich 1	Q07955	35/28	5.8	3	mRNA processing
		STIP1 homology and U box-containing protein 1	Q9UNE7	35/35	5.8	3	Chaperone activity modulation
		Heterogeneous nuclear ribonucleoproteins A2/B1	P22626	35/37	5.8	3	Pre-mRNA processinghttp://www.uniprot.org/keywords/KW-0508
		39S ribosomal protein L3, mitochondrial	P09001	35/39	5.8	2	Translation
2685	U	**Cathepsin D** ‡_(2658,2873,2674,2682,4384,4375,1968)_	P07339	28/45	5.0	20	Proteolysis
		cDNA, FLJ92320, highly similar to Homo sapiens glutathione S-transferase theta 2 (GSTT2), mRNA	B2R533	28/28	5.0	5	Transferase
		Uncharacterized protein PSME2 _(2682,2674,4384)_	A8MZ76	28/29	5.0	4	Proteasome activation
		Inositol monophosphatase	P29218	28/30	5.0	5	Phosphatidylinositol biosynthetis
		Chloride intracellular channel protein 1	O00299	28/27	5.0	3	Ion transport
		Density-regulated protein	O43583	28/22	5.0	2	Translation initiation
2879	U	**Heat shock 70 kDa protein 1 (HSP70.1)** *	P08107	25/70	5.2	4	Stress response, polypeptide folding
		A-kinase anchor protein 3	O75969	25/95	5.2	2	Capacitation and acrosome reaction
2508	U	Caspase-3 subunit p12	P42574	32/32	6.2	14	Apoptosis
		Nuclear protein Hcc-1	P82979	32/24	6.2	5	Cell growth, carcinogenesis
		Guanine nucleotide-binding protein subunit beta-2-like 1	P63244	32/35	6.2	4	Regulation of the activity of kinases, tissue differentiation
		G1/S-specific cyclin-D3	P30281	32/33	6.2	4	Cell cycle control
		ATP synthase subunit gamma, mitochondrial	P36542	32/33	6.2	3	ATP synthesis, ion transport, transport
		Carbonyl reductase [NADPH] 1	P16152	32/30	6.2	2	Catalysis of reduction
		Electron transfer flavoprotein subunit alpha, mitochondrial	P13804	32/35	6.2	2	Electron transport
		Voltage-dependent anion-selective channel protein 1	P21796	32/31	6.2	2	Apoptosis, host-virus interaction, transport
		Pyrroline-5-carboxylate reductase	A6NFM2	32/33	6.2	2	Amino-acid biosynthesis
		Cell division control protein 2 homolog	P06493	32/34	6.2	2	Cell cycle control (s-phase, mitosis)
		2,4-dienoyl-CoA reductase, mitochondrial	Q16698	32/36	6.2	2	Oxidoreductase
		Elongation factor Ts, mitochondrial	P43897	32/35	6.2	2	Protein biosynthesis
		cDNA FLJ76011, highly similar to Homo sapiens lactamase, beta 2 (LACTB2), mRNA	A8K2D6	32/33	6.2	2	Hydrolase activity

The function description and/or biological process were from the UniProt database (www.uniprot.org). Spot, spot number; FC, fold change; MW, molecular weight; pI exp, isoelectric point as determined from the 2-D gel experiments; Pep, number of unique peptides; U, unique. The number after the protein name indicated the additional spot in which the protein was found.

‡ Proteins previously reported to be used as breast cancer biomarkers and which were overexpressed in cancerous cells based on data from the literature [10], [14], [15].

*Proteins used for Q-RT-PCR validation.

**Table 2:** Mass spectrometry identification of proteins in spots up-regulated in or unique to MCF7 as compared to T47D cell line.

Spot	FC	Description	UniProt number	MW exp/pred (kDa)	pIexp	Pep	Function description and/or biological process
2808	3.2	Triosephosphate isomerase	P60174	28/27	6.2	19	Fatty acid biosynthesis, glycolysis
		Protein ETHE1, mitochondrial	O95571	28/28	6.2	2	Suppression of p53-induced apoptosis
2212	2.0	Heterogeneous nuclear ribonucleoprotein H3_(4375)_	P31942	42/37	6.5	13	mRNA processing
		Aflatoxin B1 aldehyde reductase member 2	O43488	42/40	6.5	8	Cellular aldehyde metabolism
		Glycerol-3-phosphate dehydrogenase 1-like	Q8N335	42/38	6.5	6	Glycerol-3-phosphate catabolism
		Heterogeneous nuclear ribonucleoprotein D-like	O14979	42/46	6.5	2	Transcription regulation
4384	5.2	Latexin	Q9BS40	31/26	5.5	14	Metalloendopeptidase inhibition
		Proteasome activator complex subunit 1	Q06323	31/29	5.5	4	Immunoproteasome assembly
		cDNA, FLJ96310 _(2674,2617,4375,4384)_	B2RCV1	31/33	5.5	2	Transport
4375	11	cDNA, FLJ94267, highly similar to Homo sapiens glutathione S-transferase _(3184)_	B2R983	32/28	6	16	Metabolism
		NAD-dependent deacetylase sirtuin-3, mitoch.	Q9NTG7	32/44	6	2	NAD-dependent deacetylase activity
3427	3.3	SH3 domain-binding glutamic acid-rich-like	O75368	17/13	5.2	14	Sh3 domain binding, sh3/sh2 adaptor
3184	2.7	Myosin regulatory light chain 2, nonsarcomeric	P19105	21/20	4.6	7	Cytokinesis, receptor capping, cell locomotion
3405	2.4	Profilin _(3102)_	Q4VBQ4	21/15	4.6	12	Actin binding
		Cellular retinoic acid-binding protein 1	P29762	21/16	4.6	2	Transport
		TRM112-like protein	Q9UI30	21/14	4.6	2	Protein binding
3102	2.2	**Chromobox protein homolog 3** *_(3095)_	Q13185	21/21	5.1	11	Transcription regulation
		BH3-interacting domain death agonist p11	P55957	21/22	5.1	2	Apoptosis
		Chromobox protein homolog 5	P45973	21/22	5.1	2	Repressor of transcription, chromatin assembly or disassembly
3095	7.0	**Cytochrome c-releasing factor 21** *	O75223	23/21	5.0	5	Induction of apoptosis
1968	4.7	Interleukin-10	P22301	50/21	5.0	2	Inhibits the synthesis of cytokines, B cell proliferation, anti-apoptosis
		Telomerase-binding protein EST1A	Q86US8	50/160	5.0	2	Telomere regulation, nonsense-mediated mRNA decay
		Bullous pemphigoid antigen 1, isoforms 6/9/10	O94833	50/591	5.0	2	Cell adhesion
		cAMP response element-binding protein	P16220	50/37	5.0	2	Host-virus interaction, transcription
		Coiled-coil domain-containing protein 110	Q8TBZ0	50/97	5.0	2	Unknown
		CHD9 protein	B2RTU2	50/326	5.0	2	Transcription, transcription regulation
1870	5.7	Transcriptional repressor NF-X1	Q12986	53/124	5.1	2	Transcription regulation
		MHC class I antigen (Fragment)	A2TEM8	53/11	5.1	2	Immune response
		Probable global transcription activator SNF2L2	B1ALG3	53/181	5.1	2	Transcriptional coactivator cooperating with nuclear hormone receptors
4439	U	Thioredoxin-dependent peroxide reductase, mitochondrial_(2887)_	P30048	25/28	6.2	2	Redox regulation of the cell
4492	U	Transgelin-2	P37802	20/22	5.6	8	Muscle development
4404	U	Pyridoxine 5'-phosphate oxidase (F.)	Q53FP0	29/30	5.8	5	Pyridoxine biosynthesis
		Isopentenyl-diphosphate Delta-isomerase 1	Q13907	29/26	5.8	4	Steroid, lipid, carotenoid biosynthesis
3563	U	Calcyphosin	Q13938	23/21	4.6	10	Regulation of ionic transport
4572	U	**Protein S100-A14** ‡	Q9HCY8	16/12	5.0	4	Calcium ion binding
4530	U	Calmodulin-like 5	Q5SQI3	18/16	4.2	4	Calcium ion binding
4094	U	NADP-dependent malic enzyme	P48163	75/64	5.8	24	Nadp biosynthetic process
		T-complex protein 1 subunit alpha	P17987	75/60	5.8	12	Protein folding
		Prolyl 4-hydroxylase subunit alpha-1	P13674	75/61	5.8	10	Protein metabolic process
		Heterogeneous nuclear ribonucleoprotein K	P61978	75/51	5.8	5	Host-virus interaction,mRNA processing
		Beta-galactosidase	A8MZ11	75/73	5.8	3	Glycosidase, hydrolase
		REST corepressor 1	Q9UKL0	75/53	5.8	2	transcription transcription regulation
4364	U	**Nitrilase homolog 2** *	Q9NQR4	32/31	5.9	4	Decreases the colony-forming capacity of cultured cells by arresting cells in the G2 phase of the cell cycle

The function description and/or biological process and the subcellular location were from the UniProt database (www.uniprot.org). Spot, spot number; FC, fold change; MW, molecular weight; pI exp, isoelectric point as determined from the 2-D gel experiments; Pep, number of unique peptides; U, unique. The number after the protein name indicated the additional spot in which the protein was found.

‡ Protein previously reported to be used as a breast cancer biomarker and which were overexpressed in cancerous cells based on data from the literature [9], [10].

*Protein used for Q-RT-PCR validation.

The proteomic comparisons notably led to the identification of five proteins that are used as breast cancer diagnostic and prognostic biomarkers: proliferating cell nuclear antigen (PCNA), cathepsin D, cathepsin B, protein S100-A14, and heat shock protein beta-1 (HSP27) [9], [10], [14], [15] (Table 1 and 2). PCNA was identified in a spot 6.1 times up-regulated in T47D, as compared to MCF7. Cathepsin D was found in eight different spots, whereas cathepsin B was found in a unique spot up-regulated in T47D as compared to MCF7, and protein S100-A14, in a spot unique to MCF7 as compared to T47D. HSP27 was found in four different spots: two are overexpressed in T47D and two are overexpressed in MCF7. These results showed that breast cancer biomarkers are differentially expressed in the two breast cancer cell lines.

### Comparison of protein and transcript expression

Next, we investigated the mRNA expression of proteins identified in the proteomic analyses to evaluate if there is a correlation between protein and mRNA expression. To do this, eight proteins principally involved in steroid metabolism, cell proliferation and apoptosis were selected: 17β-HSD type 10, PCNA, cathepsin B, nitrilase homolog 2, CDV3 homolog, heat shock 70 kDa protein 1 (HSP70.1), chromobox protein homolog 3 and cytochrome c-releasing factor 21. Their mRNA levels were quantified by quantitative real-time RT-PCR (Q-RT-PCR) analysis of total RNA extracts from the two cell lines T47D and MCF7. Proteomics and Q-RT-PCR data were considered to correlate if the mRNA level and protein spot were regulated in the same direction (Table 3). Except for one protein, all the other seven proteins for which the mRNA expression was evaluated, exhibited a regulation in the same direction at protein and mRNA levels in T47D as compared to MCF7. These data can indicate the existence of a semiquantitative correlation between protein and mRNA expression. Thus, it may be possible to predict the presence of a protein based on its gene expression and inversely.

**Table 3:** Q-RT-PCR values (thousand copies of mRNA/µg total RNA) of mRNAs encoding various enzymes involved in estradiol production (or action) and breast cancer cell proliferation within T47D and MCF7 and comparison with 2-D gel data.

Description	T47D	MCF7	Correlation 2-D gel and Q-RT-PCR
**Proliferating cell nuclear antigen (PCNA)** *	2,333	1,600	Yes
**Protein CDV3 homolog**	592	419	Yes
**17β-HSD type 10**	1,740	1,477	Yes
**Cathepsin B**	1,425	1,255	Yes
**Heat shock 70 kDa protein 1 (HSP70.1)**	725	1,926	No
**Chromobox protein homolog 3**	1,321	1,966	Yes
**Cytochrome c-releasing factor 21**	19	29	Yes
**Nitrilase homolog 2**	435	791	Yes
17β-HSD type 1	697	83	nd
17β-HSD type 2	N	N	nd
17β-HSD type 5	4	850	nd
17β-HSD type 7	150	159	nd
17β-HSD type 12	362	101	nd
Aromatase (P450arom)	0	N	nd
Estrogen sulfotransferase (EST)	0	0	nd
Steroid sulfatase (STS)	60	80	nd
Estrogen receptor alpha (ERα)	190	653	nd
Estrogen receptor beta (ERβ)	8	7	nd
Androgen receptor (AR)	127	96	nd

N, negligible (Q-RT-PCR values <1 thousand copies of mRNA/µg total RNA); 0, mRNA not detected after many rounds of amplification; nd, not determined. SDs were <10% of duplicates.

*Proteins in bold were selected for Q-RT-PCR validation after their identification by mass spectrometry analysis of 2-D gel spots.

In parallel work, we also measured mRNA levels of various proteins involved in estradiol (E2) synthesis, inactivation and action in the two cell lines for comparison. These proteins include several 17β-hydroxysteroid dehydrogenase (17β-HSD) enzymes and steroid receptors (Table 3). Results showed that mRNAs of 17β-HSDs types 1 and 12 and androgen receptor (AR) are expressed in greater amounts in T47D than in MCF7, whereas mRNAs of 17β-HSD type 5, estrogen receptor alpha (ERα) and steroid sulfatase are less expressed. The major difference however concerned the transcript of 17β-HSD type 5 which was 189 times lower in T47D than in MCF7. 17β-HSD type 7 was expressed at about the same level in both cell lines while mRNA levels of 17β-HSD type 2, aromatase and estrogen sulfotransferase were negligible or near zero in both cell lines (Table 3). These data show that the transcripts of enzymes and steroid receptors involved in E2 production and action are differentially expressed in the two cell lines. The correlation between protein and mRNA expression was not determined for these enzymes since they were not identified in the proteomic analyses.

## Discussion

T47D and MCF7 cells are two ER positive hormone-dependent breast cancer cell lines which additionally express AR. The two cell lines are widely used for the studies of breast cancer mechanisms. Proteomic studies of individual cell lines have been previously reported [8]–[10] but the present study established the first protein profile comparison between the two cell lines. The overlaid 2-D gel images of T47D and MCF7 showed similar spot patterns, reflecting their common origin, pleural effusion from mammary gland tumor metastasis (ATCC, www.atcc.org). Proteomic data suggest that T47D expresses a higher number of proteins than MCF7; in agreement with the understanding of cell evolution, this indicated that the first cell line could exhibit a higher number of functional mRNAs and/or more active proteins than the latter cell line.

MS analyses indicated that 18 proteins (Table 1, 2 and S1) were present in more than one spot on the gel. These proteins included heat shock protein beta-1 (implicated in stress resistance), prohibitin (implicated in cell proliferation), chromobox protein homolog 3 (implicated in transcription regulation) and cathepsin D (implicated in proteolysis). For example, cathepsin D was found in eight spots of which five were up-regulated and three were down-regulated in T47D. The presence of a protein in several spots and its regulation in different directions among the spots can be due to post-translational modifications like phosphorylation, glycosylation, or limited proteolytic cleaveage [8]. The up-regulated cathepsin D in T47D exhibited an apparent molecular weight (MW) of about 28 kDa (25, 28 and 29 kDa), whereas the down-regulated comprised a protein exhibiting a MW of 31–32 and 50 kDa. The presence of cathepsin D with several apparent MW reflects the existence of isoforms. In fact, human cathepsin D is synthesized as pre-pro-enzyme that undergoes a co-translational removal of peptide and several proteolytic cleavages to generate the pro-enzyme (a 52 kDa pro-cathepsin D), an enzymatically active 44–48 kDa intermediate and a two-chain form with non-covalently associated subunits of 14 and 31–34 kDa [8], [16], [17]. The up-regulation of the 28 kDa isoform and the down-regulation of the 50 kDa protein in T47D cells may indicate that the pro-cathepsin D is down-regulated in this cell line. This may explain why a cathepsin D with a MW higher than 33 kDa was not identified in a previous T47D and antiestrogen-resistant derivative T47D-r study [8].

Several proteins involved in cell growth and anti-apoptosis regulations and cancerogenesis are more strongly expressed in T47D than in MCF7. These proteins include caspase-3 subunit p12, Nuclear protein Hcc-1, G1/S-specific cyclin-D3, cathepsin B, protein CDV3 homolog, N(G),N(G)-dimethylarginine dimethylaminohydrolase 2 and prohibitin. Other proteins implicated in transcription repression and apoptosis regulation are, on the contrary, less abundantly expressed in T47D than in MCF7. These are chromobox protein homologs 3 and 5, BH3-interacting domain death agonist p11, cytochrome c-releasing factor 21, transcriptional repressor NF-X1, nitrilase homolog 2 and interleukin-10. From these data, it appears that proteins implicated in cell proliferation stimulation seem to be more up-regulated in T47D as compared to MCF7, whereas proteins involved in cell growth regression are therein down-regulated. Our study showed that proteins that are differentially expressed between T47D and MCF7 are implicated in all the biological functions of the cell. An example is ‘DNA replication’ which contributes to cell proliferation. In addition, markers classified as risk markers (genetic), prognostic factors (which correlate with patient outcome), predictive markers (prediction of the response or resistance to a specific therapy) and markers for the follow-up of patients with diagnosed cancer (recurrent disease detection or treatment monitoring) [9], [10], [14], [15] are found to be differentially expressed in the two cell lines. Most of these markers have been tested on tumor samples, and they are overexpressed or mutated in a significant proportion of breast cancers. The best-known molecular risk marker is PCNA [14].

Estrogens are clearly carcinogenic in humans but the molecular pathways by which these hormones induce cancer are only partially understood [18]. To improve our understanding of these pathways, the mRNA expression of enzymes implicated in E2 synthesis and action were evaluated in T47D and MCF7 cell lines. Our study shows that aromatase is not expressed in T47D and its expression in MCF7 is negligible compared to those of the 17β-HSD enzymes, indicating that E2 synthesis in these two cell lines proceeds mainly by 17β-HSD activities certainly *via* the steroid sulfatase pathway. Our results also permit a comparison of the relative mRNA expression levels of the three sex-hormone receptors, ERα, ERβ and AR, in T47D and MCF7 cells. In both cell lines, AR and ERα are more highly expressed than ERβ with ERα having the highest expression. In MCF7, AR, the cognate receptor of dihydrotestosterone (DHT), an androgen that decreases the estradiol-dependent growth of breast cancer cells [7], [19], [20], was 6.8 times less expressed than ERα, whereas the difference is only 1.5 times in T47D. This suggests that AR-mediated activities are higher in the latter cell line than in the former. DHT may thus contribute to the decrease of E2-dependent growth more efficiently in T47D cells than in MCF7 cells.

In conclusion, the present study reveals that a high number (at least 164) of proteins (including proteins involved in breast cancer cell growth regulation) are differentially expressed between the two most used human breast cancer cell lines, T47D and MCF7. This suggests that these proteins, listed in Tables 1, 2 and S1, could be differentially expressed in breast tumors. The list of differentially expressed proteins generated in the present study may provide useful information for further studies of breast cancer mechanisms with T47D and MCF7 as breast cancer cell models.

## Supporting Information

Table S1 **Additional data of mass spectrometry identification of proteins in spots up-regulated in or unique to T47D as compared to MCF7 cell line.** The function description and/or biological process were from the UniProt database (www.uniprot.org). Spot, spot number; FC, fold change; MW, molecular weight; pI exp, isoelectric point as determined from the 2-D gel experiments; Pep, number of unique peptides; U, unique. The number after the protein name indicated the additional spot in which the protein was found.
(DOC)
