# Correction: Comparison of Functional Proteomic Analyses of Human Breast Cancer Cell Lines T47D and MCF7

**DOI:** 10.1371/annotation/18f08a33-35e1-4bf9-8d21-476757dccbef

**Published:** 2012-04-26

**Authors:** Juliette Adjo Aka, Sheng-Xiang Lin

There was an error in the citation. The correct citation is: Aka JA, Lin S-X (2012) Comparison of Functional Proteomic Analyses of Human Breast Cancer Cell Lines T47D and MCF7. PLoS ONE 7(2): e31532. doi:10.1371/journal.pone.0031532 

