# Supplementary material for: Comparison of Functional Proteomic Analyses of Human Breast Cancer Cell Lines T47D and MCF7
Source: PLoS One. 2012 Feb 24;7(2):e31532. doi: 10.1371/journal.pone.0031532 (PMC3286449; doi:10.1371/journal.pone.0031532)
Supplement: Table S1 — Additional data of mass spectrometry identification of proteins in spots up-regulated in or unique to T47D as compared to MCF7 cell line. The function description and/or biological process were from the UniProt database (www.uniprot.org). Spot, spot number; FC, fold change; MW, molecular weight; pI exp, isoelectric point as determined from the 2-D gel experiments; Pep, number of unique peptides; U, unique. The number after the protein name indicated the additional spot in which the protein was found. (DOC) [file pone.0031532.s001.doc]

| **Table S1. Additional data of mass spectrometry identification of proteins in spots up-regulated or unique in T47D as compared to MCF7 cell line.** The function description and/or biological process were from the UniProt database (www.uniprot.org). Spot, spot number; FC, fold change; MW, molecular weight; pI exp, isoelectric point as determined from the 2-D gel experiments; Pep, number of unique peptides; U, unique. The number after the protein name indicated the additional spot in which the protein was found. | | | | | | | |
| --- | --- | --- | --- | --- | --- | --- | --- |
| Spot | FC | Description | UniProt number | MW exp/  pred (kDa) | pI  exp | Pep | Function description and/or  biological process |
|  |  |  |  |  |  |  |  |
| 2027 | 5.9 | Reticulocalbin-1_(1904)_ | Q15293 | 44/39 | 4.5 | 9 | Calcium-dependent activities |
|  |  | Human P37 AUF1 | Q12771 | 44/31 | 4.5 | 3 | mRNA catabolism, binding |
| 1904 | 4.2 | Actin, cytoplasmic 1 | P60709 | 47/42 | 4.5 | 7 | Cell motility |
| 2627 | 3.0 | EF-hand domain-containing protein D1 | Q9BUP0 | 30/27 | 5.2 | 30 | Calcium ion and [protein binding](http://www.ebi.ac.uk/ego/DisplayGoTerm?id=GO:0005515) |
|  |  | 3-hydroxyisobutyrate dehydrogenase, mitochondri | P31937 | 30/35 | 5.2 | 3 | [Oxidoreduction,](http://www.uniprot.org/keywords/KW-0560) [valine metabolism](http://www.ebi.ac.uk/ego/DisplayGoTerm?id=GO:0006573) |
| 1426 | 3.4 | FK506-binding protein 5 | Q13451 | 61/51 | 5.7 | 37 | [Protein folding](http://www.ebi.ac.uk/ego/DisplayGoTerm?id=GO:0006457), receptor interaction |
|  |  | Vacuolar ATP synthase subunit B, brain isoform | P21281 | 61/57 | 5.7 | 17 | [ATP synthesis; proton transport](http://www.ebi.ac.uk/ego/DisplayGoTerm?id=GO:0015986) |
|  |  | cDNA FLJ75447, highly similar to Homo sapiens peptidase D, mRNA | A8K3Z1 | 61/55 | 5.7 | 8 | [Proteolysis](http://www.ebi.ac.uk/ego/DisplayGoTerm?id=GO:0006508) |
|  |  | Tubulin beta chain | P07437 | 61/50 | 5.7 | 10 | Cell motion, |
|  |  | Uncharacterized protein WARS... | A6NGN1 | 61/49 | 5.7 | 5 | [Protein biosynthesis](http://www.uniprot.org/keywords/KW-0648) |
|  |  | Cytosolic non-specific dipeptidase | Q96KP4 | 61/53 | 5.7 | 5 | [Proteolysis](http://www.ebi.ac.uk/ego/DisplayGoTerm?id=GO:0006508) |
|  |  | Adenylyl cyclase-associated protein 2 | P40123 | 61/53 | 5.7 | 4 | [Signal transduction](http://www.ebi.ac.uk/ego/DisplayGoTerm?id=GO:0007165) |
|  |  | Translation initiation factor eIF-2B subunit gamma | Q9NR50 | 61/50 | 5.7 | 2 | [Protein biosynthesis](http://www.uniprot.org/keywords/KW-0648) |
| 2658 | 3.1 | Charged multivesicular body protein 2a_(4364)_ | O43633 | 29/25 | 5.6 | 7 | [Protein transport](http://www.uniprot.org/keywords/KW-0653), [Transport](http://www.uniprot.org/keywords/KW-0813) |
|  |  | Desmoplakin | P15924 | 29/332 | 5.6 | 5 | cytoskeleton Structural constituent |
|  |  | Junction plakoglobin | P14923 | 29/82 | 5.6 | 3 | [Cell adhesion](http://www.uniprot.org/keywords/KW-0130) |
| 2909 | 5.2 | Oligoribonuclease, mitochondrial_(2399)_ | Q9Y3B8 | 24/27 | 5.5 | 6 | Cellular nucleotide recycling |
|  |  | Ras-related protein Rab-5C | P51148 | 24/23 | 5.5 | 2 | Protein transport. vesicular traffic |
| 3226 | U | Destrin | P60981 | 19/19 | 6.0 | 8 | Actin-depolymerizing protein |
|  |  | Cofilin-1_(4492)_ | P23528 | 19/19 | 6.0 | 2 | Actin polymerization/depolymerization |
| 2873 | U | cDNA, FLJ94446, highly similar to Homo sapiens proteasome_(2658)_ | B2R9L3 | 25/29 | 5.6 | 12 | [Proteolysis](http://en.wikipedia.org/wiki/Proteolysis) |
|  |  | Brain type mu-glutathione S-transferase_(3095)_ | A4UJ43 | 25/25 | 5.6 | 5 | [Glutathione transfert](http://www.ebi.ac.uk/ego/DisplayGoTerm?id=GO:0004364) |
|  |  | Proteasome subunit beta type-4 | P28070 | 25/29 | 5.6 | 2 | [Proteolysis](http://en.wikipedia.org/wiki/Proteolysis) |
| 2887 | U | Glutathione S-transferase theta-2 | P30712 | 25/28 | 6.2 | 14 | [Glutathione transfert](http://www.ebi.ac.uk/ego/DisplayGoTerm?id=GO:0004364) |
|  |  | Proteasome subunit beta type-3 | P49720 | 25/23 | 6.2 | 6 | Proteolysis |
|  |  | NADH dehydrogenase [ubiquinone] flavoprotein 2, mitochondrial | P19404 | 25/27 | 6.2 | 6 | [Electron transport](http://www.uniprot.org/keywords/KW-0249) |
|  |  | cDNA, FLJ93804, highly similar to Homo sapiens gp25L2 protein_(2869)_ | B2R8A2 | 25/25 | 6.2 | 4 | Transport |
|  |  | Proteasome subunit alpha type-2 | P25787 | 25/26 | 6.2 | 4 | Proteolysis |
|  |  | Ras-related protein Rab-7a | P51149 | 25/23 | 6.2 | 3 | [Protein transport](http://www.uniprot.org/keywords/KW-0653), [transport](http://www.uniprot.org/keywords/KW-0813) |
|  |  | Beta-hexosaminidase subunit beta chain A | P07686 | 25/63 | 6.2 | 2 | Degradation of gm2 gangliosides |
|  |  | StAR-related lipid transfer protein 5 | Q9NSY2 | 25/24 | 6.2 | 3 | Intracellular sterol/lipid transport |
| 3009 | U | Molybdenum cofactor synthesis protein 2 large subunit | O96007 | 23/21 | 5.2 | 8 | [Molybdenum cofactor biosynthesis](http://www.uniprot.org/keywords/KW-0501) |
|  |  | RAB1B protein | Q6FIG4 | 23/22 | 5.2 | 3 | [Protein transport](http://www.ebi.ac.uk/ego/DisplayGoTerm?id=GO:0015031), GTPase mediated signal transduction |
|  |  | cDNA FLJ75516, highly similar to Xenopus tropicalis ubiquitin C, mRNA | A8K674 | 23/68 | 5.2 | 3 | [Protein modification process](http://www.ebi.ac.uk/ego/DisplayGoTerm?id=GO:0006464) |
|  |  | cDNA, FLJ92406, highly similar to Homo sapiens histone 1, H2ag (HIST1H2AG), mRNA | B2R5B3 | 23/14 | 5.2 | 2 | [Nucleosome assembly](http://www.ebi.ac.uk/ego/DisplayGoTerm?id=GO:0006334) |
|  |  | Heme-binding protein 1 | Q9NRV9 | 23/21 | 5.2 | 2 | [Circadian rhythm](http://www.ebi.ac.uk/ego/DisplayGoTerm?id=GO:0007623) |
| 2674 | U | RNA polymerase II subunit A C-terminal domain phosphatase SSU72 | Q9NP77 | 28/23 | 5.3 | 3 | [mRNA processing](http://www.uniprot.org/keywords/KW-0507) |
|  |  | cDNA FLJ78235 | A8K2H4 | 28/38 | 5.3 | 2 | [Proteolysis](http://www.ebi.ac.uk/ego/DisplayGoTerm?id=GO:0006508), [regulation of catalytic activity](http://www.ebi.ac.uk/ego/DisplayGoTerm?id=GO:0050790) |
